# *IL1RL1* polymorphisms rs12479210 and rs1420101 are associated with increased lung cancer risk in the Chinese Han population

**DOI:** 10.3389/fgene.2023.1183528

**Published:** 2023-08-31

**Authors:** Qi Li, Chan Zhang, Yujing Cheng, Xin Yang, Wanlu Chen, Kunhua He, Mingwei Chen

**Affiliations:** ^1^ Department of Respiratory Medicine, The First Affiliated Hospital of School of Medicine of Xi’an Jiaotong University, Xi’an, Shaanxi, China; ^2^ Department of Blood Transfusion, The First People’s Hospital of Yunnan Province, The Affiliated Hospital of Kunming University of Science and Technology, Kunming, China; ^3^ Department of Blood Transfusion, Qujing No. 1 Hospital, Qujing, China

**Keywords:** IL1RL1, genetic, polymorphism, lung cancer, case-control study

## Abstract

**Background:** Lung cancer is one of the most common human malignant diseases. In this study, we aimed to explore the association between *IL1RL1* genetic polymorphisms and lung cancer risk in the Chinese Han population.

**Methods:** We selected and genotyped six SNPs in the *IL1RL1* gene using the Agena MassARRAY system in 507 lung cancer patients and 507 healthy controls. The association between *IL1RL1* variants and lung cancer risk was assessed using logistic regression to calculate odds ratios (ORs) and 95% confidence intervals (CIs). Multi-factor dimensionality reduction (MDR) was used to analyze the impact of SNP-SNP interactions on the risk of lung cancer.

**Results:** The results of overall analysis indicated that rs12479210 (T vs. C: OR = 1.42, FDR-*p* = 0.002; TC vs. CC: OR = 1.70, FDR-*p* < 0.0001; TT vs. CC: OR = 1.77, FDR-*p* = 0.032; TT-TC vs. CC: OR = 1.71, FDR-*p* = 0.001; additive: OR = 1.44, FDR-*p* = 0.001) and rs1420101 (T vs. C: OR = 1.31, FDR-*p* = 0.036; TT-TC vs. CC: OR = 1.42, FDR-*p* = 0.031; additive: OR = 1.30, FDR-*p* = 0.030) were associated with an increased the risk of lung cancer among the Chinese Han population. Stratified analysis also found the association between these two SNPs and lung cancer risk. However, there were no significant association observed between the other four SNPs (rs3771180, rs3771175, rs10208293, and rs10197862) in *IL1RL1* and lung cancer risk. Furthermore, MDR analysis showed that rs12479210 was the best single model with the highest testing accuracy (0.566) and perfect CVC (10/10) for predicting lung cancer risk. The expression level of the *IL1RL1* gene is lower in lung cancer tissue than normal tissue, and there are significant differences in the expression levels of *IL1RL1* between rs12479210 and rs1420101 genetypes in lung cancer tissue (*p* < 0.05).

**Conclusion:** Our findings suggest that *IL1RL1* genetic variants (rs12479210 and rs1420101) are associated with an increased lung cancer risk in the Chinese Han population. These risk variants may serve as biomarkers for the prevention and treatment of lung cancer.

## Introduction

The incidence and mortality rates of lung cancer are increasing globally (Bade and Dela Cruz, 2020). In 2020, lung cancer was the second most commonly diagnosed cancer and the leading cause of cancer death, with an estimated 2.2 million new cancer cases (11.4%) and 1.8 million deaths (18.0%) (Sung et al., 2021). The incidence and mortality rates of lung cancer are approximately two times higher in men than in women. In China, lung cancer is the leading cause of death in both males and females, with an estimated 870,982 new cases and 766,898 deaths expected in 2022 (Xia et al., 2022). Lung cancer is a complex pathological process influenced by multiple factors, including cigarette smoking, which is reported in about one-third of adults worldwide and has a strong relationship between cigarette smoke exposure and lung cancer has been proven (Taucher et al., 2022). Other nontobacco risk factors include poor diet, occupational exposures, air pollution, chronic lung disease, and lung infections (Malhotra et al., 2016; Huang et al., 2021; Wei et al., 2021; Moayedi-Nia et al., 2022). Despite recent advances in treatments, lung cancer remains a disease with a poor prognosis, and it is often not diagnosed until the cancer is at an advanced stage. Therefore, early diagnosis of lung cancer is crucial. Genetic factors have been shown to play a crucial role in the development of lung cancer. In a Spanish study describing the characteristics of female lung cancer patients, 42.5% of patients had a family history of tumors (Garrido et al., 2019). Single nucleotide polymorphisms (SNPs) of candidate genes have also been found to be associated with lung cancer risk (Khadhraoui et al., 2020; Chongtham et al., 2022; Zhang et al., 2022).

The Interleukin 1 receptor like 1 (*IL1RL1*) gene, also known as *ST2*, *ST2L*, and *IL33R*, encodes a protein that belongs to the family of IL1R, and it is only known ligand is *IL-33* (13). The *IL-33*/*ST2L* signal induces transcription of downstream inflammatory and anti-inflammatory genes by activating diverse intracellular kinases and factors to mount an adequate immune response (14). The *IL-33*/*ST2* axis affects tumor growth by regulating mitophagy in macrophages and reprogramming their polarization (15). Xu et al. (16) demonstrated that the *IL33*-*IL1RL1* pathway influenced tumor growth by regulating autophagy and reprogramming of macrophages. *ST2* was significantly downregulated in human lung cancer tissues and cells compared to normal lung tissues and cells (17). Furthermore, Wang et al. (18) showed that the *IL33*-*IL1RL1* signaling pathway is involved in the growth and metastasis of lung cancer. However, little is known about the correlation between *IL1RL1* polymorphisms and lung cancer.

Therefore, in this study we aimed to assess the possible association between six *IL1RL1* gene polymorphisms (rs12479210, rs3771180, rs1420101, rs3771175, rs10208293, and rs10197862) and susceptibility to lung cancer in the Chinese Han population. This research may provide a new biomarker for the prevention and development of lung cancer.

## Materials and methods

### Study population

The sample size of this study was estimated using G*Power (3.1.9.7) software with the following parameters: tails = two, effect size = 0.2, *a* = 0.05, power = 0.889, and allocation ratio = 1. A total of 1014 participants were randomly enrolled from Xuanwei city, including 507 newly histologically diagnosed lung cancer patients in the case group. Patients who underwent chemotherapy, surgery, radiotherapy, had other tumors or inflammatory diseases were excluded. The control group consisted of 507 healthy individuals recruited from medical examination centers, with no history of cancers, lung dysfunction, or related diseases. Basic information, such as age, gender, body mass index (BMI), smoking and drinking status, and clinical test indicators (tumor type and stage), were collected from questionnaires and clinical data.

### SNPs selection and genotyping

First, we searched the physical location (2:102928023-102968497) of the *IL1RL1* gene (GRCh37. p13) through the NCBI database (https://www.ncbi.nlm.nih.gov/gene/). Second, we downloaded ped and info files of mutation sites in *IL1RL1* in the Chinese Han Beijing (CHB) population from 1000 Genome Project through VCF to PED Converter window (http://grch37.ensembl.org/Homo Sapiens/Tools/VcftoPed), and we used Haploview software to screen SNPs with Hardy-Weinberg equilibrium (HWE) > 0.05, minor allele frequency (MAF) > 0.05, and min genotype frequency >75%, *r* ^2^ > 0.8. Finally, based on previous studies associated with respiratory diseases (Ferreira et al., 2011; Shrine et al., 2019; Sun et al., 2019; Wu et al., 2021; Li et al., 2022; Rojo-Tolosa et al., 2023) and primer design, a total of six SNPs in the *IL1RL1* gene were randomly selected (Supplementary Table S1). The potential role of SNPs was predicted using RegulomeDB (https://regulomedb.org/), HaploReg (https://pubs.broadinstitute.org/mammals/haploreg/haploreg.php), and Genotype-Tissue Expression (GTEx) Portal databases (https://gtexportal.org). Peripheral blood samples were collected from each subject, and genomic DNA was extracted using the GoldMag DNA extraction Kit (GoldMag Co. Ltd., Xi’an, China) according to the manufacturer’s instructions. The concentration and purity of DNA were measured by a spectrophotometer (NanoDrop 2000; Thermo Fisher Scientific, Waltham, MA, USA), with an OD260/OD280 ratio between 1.7 and 2.0 and a concentration greater than 20 ng/μL. The primers for polymerase chain reaction (PCR) and unique base extension for the six SNPs in *IL1RL1* were designed by Agena Bioscience Assay Design Suite version 2.0 software and synthesized by Bioengineering (Shanghai Co., Ltd.). The primer sequence is shown in Table 1. Genotyping of *IL1RL1* variants was conducted by the Agena MassARRAY system (Agena Bioscience, San Diego, CA, USA), and data management and analysis were performed using AgenaTyper 4.0 software.

**TABLE 1:** Primer sequences of PCR and unique base extension.

SNP-ID	1st-PCRP	2nd-PCRP	UEP-SEQ
rs12479210	ACG​TTG​GAT​GTG​TGG​TAC​AAC​CAC​TAA​CTC	ACG​TTG​GAT​GGG​GAC​TTC​ATG​TTA​ATG​GG	TAA​TGG​GTA​TGG​GGT​TAT​ACT
rs3771180	ACG​TTG​GAT​GGG​CCA​AAT​CTA​TGA​CTT​GTT​C	ACG​TTG​GAT​GTT​CCT​CTC​AAG​GGA​TTA​CTC	cAC​ATC​AAG​AAT​TCT​TAG​TAC​ATG​AT
rs1420101	ACG​TTG​GAT​GTT​TGG​TGT​CAG​AGT​TTC​TGC	ACG​TTG​GAT​GGT​ATA​CCA​TCA​CAA​AGC​CTC	cTC​ACA​AAG​CCT​CTC​ATT​A
rs3771175	ACG​TTG​GAT​GGA​ACG​GAC​CAT​GCT​TAC​AAC	ACG​TTG​GAT​GGT​GTC​ACT​GTA​TGT​GAA​AGG	ggg​cAT​GCA​CCA​ACA​ACC​G
rs10208293	ACG​TTG​GAT​GAG​AGA​AGA​GTT​CAC​AAG​GAG	ACG​TTG​GAT​GAT​CAC​AAC​TCC​TGT​GTT​TGC	act​taA​TAC​ATT​TAT​GTC​CAG​TAC​CT
rs10197862	ACG​TTG​GAT​GCT​GGG​AAT​GCT​AAT​AGC​CTC	ACG​TTG​GAT​GGA​AGA​AGA​GAG​CCT​TGA​GAG	AAT​TGT​ATC​TTT​CAC​AAG​TCT​C

SNP, single nucleotide polymorphism; 1st, frist; 2nd, second; PCRP, polymerase chain reaction primer; UEP-SEQ, unique base extension primer sequence.

### Bioinformatics analysis

The differences between six SNPs in *IL1RL1* and the expression level of *IL1RL1* in lung cancer tissues were analyzed using GTEx Portal database (https://gtexportal.org/). The expression level of *IL1RL1* in normal and lung adenocarcinoma (LUAD) and lung squamous cell carcinoma (LUSC) tissues was analyzed using GEPIA online analysis software based on TCGA database (http://gepia.cancer-pku.cn/). We predicted the signaling pathways involved in the *IL1RL1* gene through the PathCards database (https://pathcards.genecards.org/).

### Statistical analysis

Statistical analysis was performed using IBM SPSS software (version 25.0, SPSS Inc., Chicago, Illinois, USA) and PLINK software (version 1.07). The distribution of age and gender in the case and control group was evaluated using Student’s t-test and Pearson’s chi-square test, respectively. The *p*-value of HWE in controls was calculated by the chi-square test. The association between *IL1RL1* variants and lung cancer risk was assessed using odds ratio (OR) and 95% confidence interval (95% CI) calculated by logistic regression under multiple genetic models (allele, codominant, dominant, recessive, and additive). To reduce the influence of confounding factors (age, gender, and smoking status) on the statistical results, stratified analyses were performed, and forest plots of stratified results were drawn using Sangerbox software (version 3.0). The Akaike Information Criterion (AIC) and Bayesian Information Criterion (BIC) values were calculated using SNPStats software to select the optimal model. False discovery rate (FDR) correction (q = *p* *(n/k); n is the total number of *p*-values; k is the order in which *p*-values are sorted from smallest to largest) was performed for *p*-values to reduce false positives in the results. The multifactor dimensionality reduction (MDR) method was used to further evaluate the effect of SNP-SNP interactions on lung cancer risk by MDR 3.0.2 software. To determine the reliability of significant correlation results, statistical power and false positive report probability (FPRP) values of SNPs were calculated using the Excel spreadsheet offered on Wacholder’s website (Wacholder et al., 2004). All statistical analyses were two-sided, and *p* < 0.05 was considered statistically significant.

## Results

### Participant characteristics

Table 2 describes the basic characteristics of the study participants. This study included 507 patients diagnosed with lung cancer (average age: 61.12 ± 10.03 years, 350 males and 157 females) and 507 healthy controls (average age: 60.97 ± 9.25 years, 349 males and 158 females). The results showed that the control and case groups were well-matched in terms of age (*p* = 0.810) and gender (*p* = 0.946) distribution. However, there were significant differences in the distribution of BMI, smoking, and alcohol consumption between the case group and the control group (*p <* 0.05). Therefore, in order to reduce the impact of these confounding factors on association analysis results, we conducted correction and stratified analysis.

**TABLE 2:** Basic characteristics of participants.

Variables		Cases (n = 507)	Control (n = 507)	*p*-value
Age (years)	Mean ± SD	61.12 ± 10.03	60.97 ± 9.25	0.810
	>60	280 (55.2%)	295 (58.2%)	0.342
	≤60	227 (44.8%)	212 (41.8%)	
Gender	Male	350 (69.0%)	349 (68.8%)	0.946
	Female	157 (31.0%)	158 (31.2%)	
BMI (kg/m2)	Mean ± SD	22.79 ± 3.47	24.15 ± 3.07	
	>24	139 (27.4%)	146 (28.8%)	<0.001
	≤24	355 (70.0%)	189 (37.3%)	
	Missing	13 (2.6%)	172 (33.9%)	
Drinking	Yes	113 (22.3%)	118 (23.3%)	<0.001
	No	357 (70.4%)	174 (34.3%)	
	Missing	37 (7.3%)	215 (42.4%)	
Smoking	Yes	251 (49.5%)	129 (25.4%)	<0.001
	No	250 (49.3%)	189 (37.3%)	
	Missing	6 (1.2%)	189 (37.3%)	
Types of LC	SCLC	58 (11.4%)		
	SCC	137 (27.0%)		
	Adenocarcinoma	189 (37.3%)		
	Missing	123 (24.3%)		
Tumor stage	I-II	88 (20.0%)		
	III-IV	277 (54.6%)		
	Missing	142 (25.4%)		

SD, standard deviation; BMI, body mass index; SCC, squamous cell carcinoma; SCLC, small cell lung cancer.

*p*-value was calculated by Student’s t-test for age and *χ* ^2^ For gender.

*p* < 0.05 indicates statistical significance.

### Basic information of SNPs in *IL1RL1*

This research selected six SNPs (rs12479210, rs3771180, rs1420101, rs3771175, rs10208293, and rs10197862) in the *IL1RL1* gene as candidate SNPs. As shown in Table 3, rs3771175 is located in the 3′-UTR of *IL1RL1* gene, and the rest loci are located in the intron region. RegulomeDB and HaploReg analysis predicted the potential functions of these SNPs. The call rates of all SNPs genotype were greater than 95%, and were consistent with HWE, indicating that our samples satisfied random distribution and the SNP genotyping technique was reliable (*p* > 0.05, Table 3).

**TABLE 3:** Basic information and HWE of six SNPs in *IL1RL1*.

SNP-ID	Chr	Position	Functional consequence	Allele (A/B)	MAFcase	MAF control	HWE-*p*	RegulomeDB rank	HaploReg
rs12479210	2	102332701	Intron	T/C	0.389	0.309	0.754	Other	Motifs changed; GRASP QTLhits; Selected eQTL hits
rs3771180	2	102337157	Intron	T/G	0.084	0.091	0.592	TF binding + any motif + DNase Footprint + DNase peak	Promoter histone marks; Enhancer histone marks; DNAse; Proteins bound; Motifs changed; NHGRI/EBI GWAS hits; GRASP QTL hits; Selected eQTL hits
rs1420101	2	102341256	Intron	T/C	0.369	0.309	0.349	TF binding + any motif + DNase peak	Enhancer histone marks; Motifs changed; NHGRI/EBI GWAS hits; GRASP QTL hits; Selected eQTL hits
rs3771175	2	102343750	3′-UTR	A/T	0.078	0.079	0.540	TF binding + DNase peak	Enhancer histone marks; Motifs changed; NHGRI/EBI GWAS hits; GRASP QTL hits; Selected eQTL hits
rs10208293	2	102349850	Intron	A/G	0.134	0.139	0.854	Other	Enhancer histone marks; Selected eQTL hits
rs10197862	2	102350089	Intron	G/A	0.088	0.091	0.587	TF binding or DNase peak	NHGRI/EBI GWAS hits; GRASP QTL hits; Selected eQTL hits

SNP, single nueleotide polymorphism; Chr, Chromosome; MAF, minor allele frequency; HWE, hardy-weinberg equilibrium; eQTL, expression quantitative trait locus; QTL, quantitative trait locus; TF, transcription factor; GRASP, genome-wide repository of associations between SNPs, and phenotypes; NHGRI, national human genome research institute; EBI, european bioinformatics institute; GWAS, genome-wide association study.

*p* < 0.05 indicates statistical significance.

### Association between *IL1RL1* polymorphisms and lung cancer risk (overall analysis)

Table 4 presents the correlation between *IL1RL1* variants and lung cancer susceptibility in multiple genetic models. The results showed that rs12479210 was associated with an increased risk of lung cancer under the allele (T vs. C: OR = 1.42, 95% CI: 1.18-1.71, *p* = 0.0001, FDR-*p* = 0.002), codominant (TC vs. CC: OR = 1.70, 95% CI: 1.30-2.22, *p* < 0.0001, FDR-*p* < 0.0001; TT vs. CC: OR = 1.77, 95% CI: 1.17-2.69, *p* = 0.007, FDR-*p* = 0.032), dominant (TT-TC vs. CC: OR = 1.71, 95% CI: 1.33-2.21, *p* = 0.0001, FDR-*p* = 0.001), and additive models (OR = 1.44, 95% CI: 1.20-1.75, *p* = 0.0001, FDR-*p* = 0.001). The dominant model was the optimal model for rs12479210 with the minimum AIC (1393.2) and BIC (1412.9) values. Additionally, rs1420101 was significantly associated with an increased risk of lung cancer under the allele (T vs. C: OR = 1.31, 95% CI: 1.09-1.57, *p* = 0.005, FDR-*p* = 0.036), dominant (TT-TC vs. CC: OR = 1.42, 95% CI: 1.10-1.82, *p* = 0.006, FDR-*p* = 0.031), and additive models (OR = 1.30, 95% CI: 1.08-1.56, *p* = 0.005, FDR-*p* = 0.030). The additive model was the optimal model for rs1420101 with the minimum AIC (1404.5) and BIC (1424.1) values. Furthermore, the statistical power and FPRP of rs12479210 and rs1420101 were analyzed under multiple genetic models (Table 5), and the results showed that the correlation between these two SNPs and lung cancer risk was reliable. However, there were no significant differences between the other four SNPs (rs3771180, rs3771175, rs10208293, and rs10197862) in *IL1RL1* and lung cancer risk.

**TABLE 4:** Associations between *IL1RL1* polymorphisms and LC risk under multiple genetic models.

SNP-ID	Model	Genotype	Case (%)	Control (%)	OR (95% CI)	*p*	FDR-*p*	AIC	BIC
rs12479210	Allele	C	620 (61.1)	698 (69.1)	1				
		T	394 (38.9)	312 (30.9)	1.42 (1.18–1.71)	0.0001	0.002		
	Codominant	CC	178 (35.1)	243 (48.1)	1			1395.2	1419.8
		TC	264 (52.1)	212 (42)	1.70 (1.30–2.22)	<0.0001	<0.0001		
		TT	65 (12.8)	50 (9.9)	1.77 (1.17–2.69)	0.007	0.032		
	Dominant	CC	178 (35.1)	243 (48.1)	1			1393.2	1412.9
		TT-TC	329 (64.9)	262 (51.9)	1.71 (1.33–2.21)	0.0001	0.001		
	Recessive	TC-CC	442 (87.2)	455 (90.1)	1			1408.7	1428.4
		TT	65 (12.8)	50 (9.9)	1.34 (0.90–1.98)	0.144	0.432		
	Additive	---	---	---	1.44 (1.20–1.75)	0.0001	0.001	1396.2	1415.8
rs3771180	Allele	G	929 (91.6)	918 (90.9)	1				
		T	85 (8.4)	92 (9.1)	0.91 (0.67–1.24)	0.563	0.965		
	Codominant	GG	424 (83.6)	418 (82.8)	1			1411.5	1436.1
		TG	81 (16)	82 (16.2)	0.98 (0.70–1.37)	0.887	1.101		
		TT	2 (0.4)	5 1)	0.39 (0.08–2.03)	0.264	0.731		
	Dominant	GG	424 (83.6)	418 (82.8)	1			1410.7	1430.4
		TT-TG	83 (16.4)	87 (17.2)	0.94 (0.68–1.31)	0.723	1.085		
	Recessive	TG-GG	505 (99.6)	500 (99)	1			1409.5	1429.2
		TT	2 (0.4)	5 1)	0.39 (0.08–2.04)	0.266	0.684		
	Additive	---	---	---	0.91 (0.67–1.25)	0.566	0.926	1410.5	1430.2
rs1420101	Allele	C	640 (63.1)	699 (69.1)	1				
		T	374 (36.9)	313 (30.9)	1.31 (1.09–1.57)	0.005	0.036		
	Codominant	CC	203 (40)	246 (48.6)	1			1406.1	1430.7
		TC	234 (46.2)	207 (40.9)	1.37 (1.05–1.78)	0.020	0.080		
		TT	70 (13.8)	53 (10.5)	1.60 (1.07–2.39)	0.022	0.079		
	Dominant	CC	203 (40)	246 (48.6)	1			1404.7	1424.4
		TT-TC	304 (60)	260 (51.4)	1.42 (1.10–1.82)	0.006	0.031		
	Recessive	TC-CC	437 (86.2)	453 (89.5)	1			1409.6	1429.3
		TT	70 (13.8)	53 (10.5)	1.37 (0.94–2.00)	0.105	0.344		
	Additive	---	---	---	1.30 (1.08–1.56)	0.005	0.030	1404.5	1424.1
rs3771175	Allele	T	935 (92.2)	932 (92.1)	1				
		A	79 (7.8)	80 (7.9)	0.98 (0.71–1.36)	0.924	1.040		
	Codominant	TT	430 (84.8)	430 (85)	1			1413.5	1438.1
		AT	75 (14.8)	72 (14.2)	1.04 (0.74–1.48)	0.810	1.122		
		AA	2 (0.4)	4 (0.8)	0.50 (0.09–2.74)	0.422	0.844		
	Dominant	TT	430 (84.8)	430 (85)	1			1412.2	1431.9
		AA-AT	77 (15.2)	76 (15)	1.02 (0.72–1.43)	0.932	0.959		
	Recessive	AT-TT	505 (99.6)	502 (99.2)	1			1411.6	1431.2
		AA	2 (0.4)	4 (0.8)	0.49 (0.09–2.72)	0.418	0.885		
	Additive	---	---	---	0.99 (0.71–1.36)	0.931	1.016	1412.2	1431.9
rs10208293	Allele	G	878 (86.6)	864 (86.1)	1				
		A	136 (13.4)	140 (13.9)	0.96 (0.74–1.23)	0.728	1.048		
	Codominant	GG	377 (74.4)	372 (74.1)	1			1407.5	1432.1
		AG	124 (24.5)	120 (23.9)	1.02 (0.76–1.36)	0.897	1.076		
		AA	6 (1.2)	10 2)	0.59 (0.21–1.63)	0.306	0.689		
	Dominant	GG	377 (74.4)	372 (74.1)	1			1406.6	1426.3
		AA-AG	130 (25.6)	130 (25.9)	0.99 (0.74–1.31)	0.921	1.070		
	Recessive	AG-GG	501 (98.8)	492 (98)	1			1405.5	1425.2
		AA	6 (1.2)	10 2)	0.58 (0.21–1.62)	0.301	0.722		
	Additive	---	---	---	0.95 (0.74–1.23)	0.717	1.122	1406.5	1426.1
rs10197862	Allele	A	925 (91.2)	913 (90.9)	1				
		G	89 (8.8)	91 (9.1)	0.97 (0.71–1.31)	0.821	1.095		
	Codominant	AA	421 (83)	416 (82.9)	1			1408.1	1432.7
		GA	83 (16.4)	81 (16.1)	1.02 (0.73–1.42)	0.931	0.986		
		GG	3 (0.6)	5 1)	0.59 (0.14–2.49)	0.473	0.851		
	Dominant	AA	421 (83)	416 (82.9)	1			1406.7	1426.3
		GG-GA	86 (17)	86 (17.1)	0.99 (0.71–1.38)	0.953	0.953		
	Recessive	AA-GA	504 (99.4)	497 (99)	1			1406.1	1425.8
		GG	3 (0.6)	5 1)	0.59 (0.14–2.48)	0.471	0.892		
	Additive	---	---	---	0.97 (0.71–1.31)	0.830	1.067	1406.6	1426.3

LC, lung cancer; SNP, single nucleotide polymorphism; OR, odds ratio; CI, confidence interval; FDR, false discovery rate AIC, akaike information criterion; BIC, Bayesian Information Criterion.

OR, and 95% CI, were calculated using logistic regression adjusted with age and gender.

*p* < 0.05 indicate statistical significance.

**TABLE 5:** FPRP of the association *IL1RL1* polymorphisms and LC risk.

SNP-ID	Model	Statistical power	Prior probability				
			0.25 (b)	0.1 (b)	0.01	0.001	0.0001
rs12479210	Allele	0.718	0.001	0.003	0.029^b^	0.232	0.751
	Homozygote	0.716	0.030	0.086	0.509	0.913	0.991
	Heterozygote	0.884	0.000	0.001	0.011^b^	0.099^b^	0.524
	Dominant	0.884	0.000	0.000	0.005^b^	0.045^b^	0.319
	Additive	1.000	0.001	0.002	0.024^b^	0.198^b^	0.712
rs1420101	Allele	0.929	0.011	0.032	0.270	0.788	0.974
	Homozygote	0.862	0.070	0.185	0.714	0.962	0.996
	Heterozygote	0.998	0.053	0.143	0.647	0.949	0.995
	Dominant	0.997	0.017	0.048	0.358	0.849	0.983
	Additive	0.938	0.015	0.044	0.336	0.836	0.981

FPRP, false positive report probability; SNP, single nucleotide polymorphism; OR, odds ratio; CI, confidence interval.

The level of false positive report probability threshold was set at 0.2 and noteworthy findings are presented.

### Stratified analysis of the association between *IL1RL1* SNPs and lung cancer risk

**FIGURE 1:**
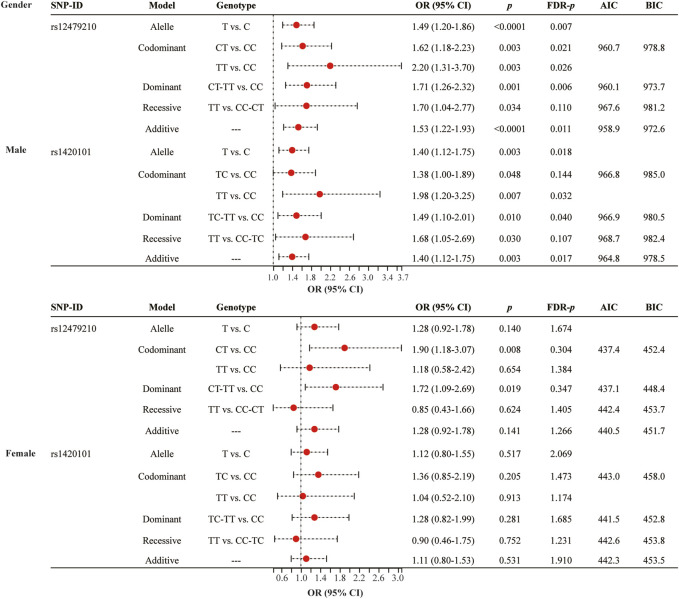
Association between rs12479210 and rs1420101 and lung cancer risk stratified by gender. SNP, single nucleotide polymorphism; OR, odds ratio; CI, confidence interval; FDR, false discovery rate; AIC, Akaike Information Criterion; BIC, Bayesian Information Criterion; *p* < 0.05 indicates statistical significance.

To reduce the impact of confounding factors on the analysis results, we conducted stratified analysis based on mean age (>60 and ≤60), gender (male and female), and smoking status (yes and no). Gender stratified analysis (Figure 1 and Supplementary Table S2) showed that rs12479210 (T vs. C: OR = 1.49, 95% CI: 1.20-1.86, *p* < 0.0001, FDR-*p* = 0.007; CT vs. CC: OR = 1.62, 95% CI: 1.18-2.23, *p* = 0.003, FDR-*p* = 0.021; TT vs. CC: OR = 2.20, 95% CI: 1.31-3.70, *p* = 0.003, FDR-*p* = 0.026; CT-TT vs. CC: OR = 1.71, 95% CI: 1.26-2.32, *p* = 0.001, FDR-*p* = 0.006; additive: OR = 1.53, 95% CI: 1.22-1.93, *p* < 0.0001, FDR-*p* = 0.011) and rs1420101 (T vs. C: OR = 1.40, 95% CI: 1.12-1.75, *p* = 0.003, FDR-*p* = 0.018; TT vs. CC: OR = 1.98, 95% CI: 1.20-3.25, *p* = 0.007, FDR-*p* = 0.032; TC-TT vs. CC: OR = 1.49, 95% CI: 1.10-2.01, *p* = 0.010, FDR-*p* = 0.040; additive: OR = 1.40, 95% CI: 1.12-1.75, *p* = 0.003, FDR-*p* = 0.017) were associated with an increased risk of lung cancer in males. The dominant model and the additive model were the optimal models for rs12479210 (AIC = 960.1 and BIC = 973.7) and rs1420101 (AIC = 964.8 and BIC = 978.5) with the minimum AIC and BIC values, respectively. However, no significant association was found between *IL1RL1* polymorphisms (rs12479210 and rs1420101) and the risk of lung cancer in females.

**FIGURE 2:**
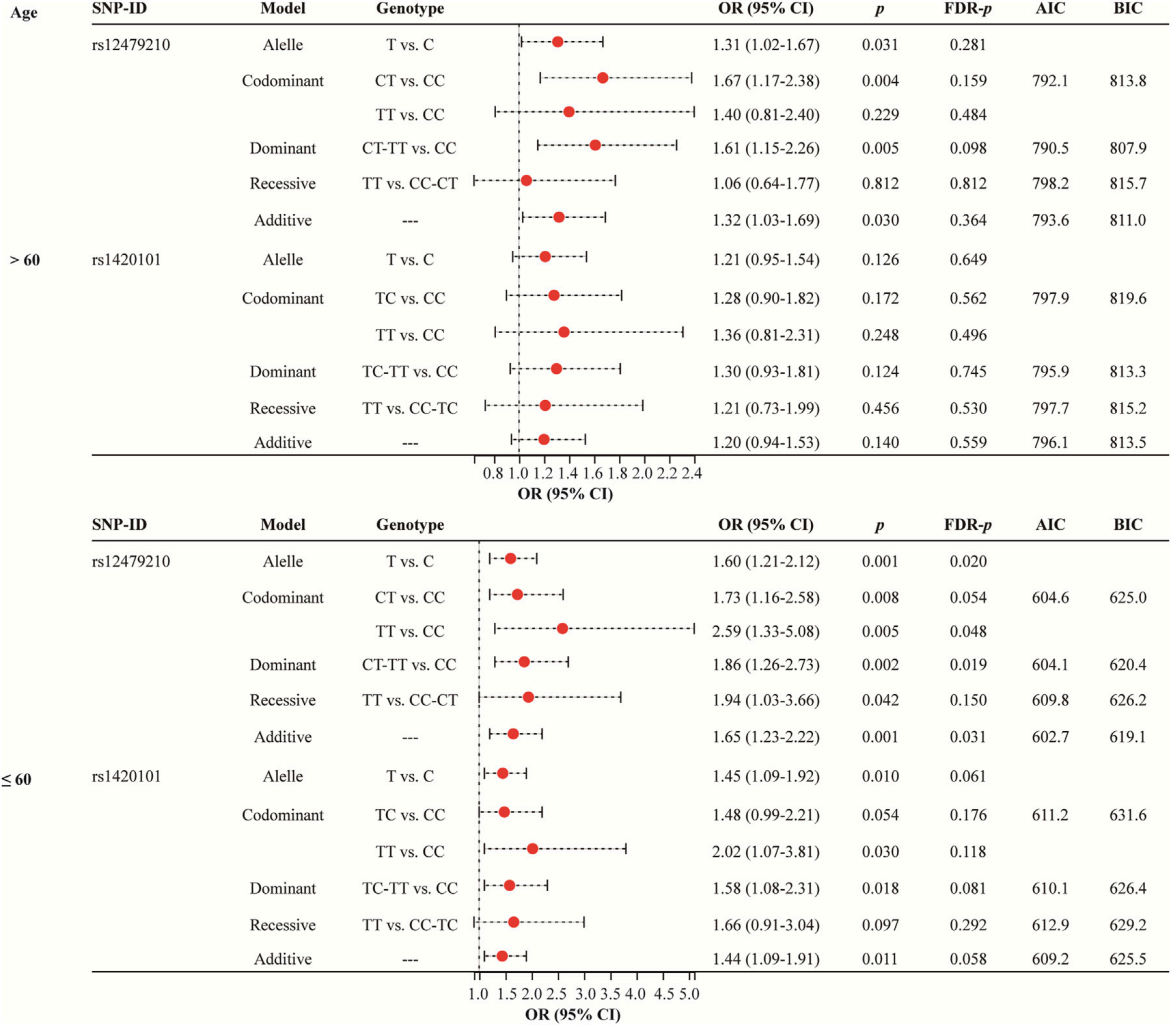
Association between rs12479210 and rs1420101 and lung cancer risk stratified by mean age. SNP, single nucleotide polymorphism; OR, odds ratio; CI, confidence interval; FDR, false discovery rate; AIC, Akaike Information Criterion; BIC, Bayesian Information Criterion; *p* < 0.05 indicates statistical significance.

Age stratified analysis (Figure 2 and Supplementary Table S2) revealed that rs12479210 (T vs. C: OR = 1.60, 95% CI: 1.21-2.12, *p* = 0.001, FDR-*p* = 0.020; TT vs. CC: OR = 2.59, 95% CI: 1.33-5.08, *p* = 0.005, FDR-*p* = 0.048; CT-TT vs. CC: OR = 1.86, 95% CI: 1.26-2.73, *p* = 0.002, FDR-*p* = 0.019; additive: OR = 1.65, 95% CI: 1.23-2.22, *p* = 0.001, FDR-*p* = 0.031) was associated with an increased risk of lung cancer in participants aged >60. The additive model was the optimal model for rs12479210 with the minimum AIC (602.7) and BIC (619.1) values in participants aged >60. However, no significant association was found between IL1RL1 polymorphisms (rs12479210 and rs1420101) and the risk of lung cancer in participants aged ≤60.

**FIGURE 3:**
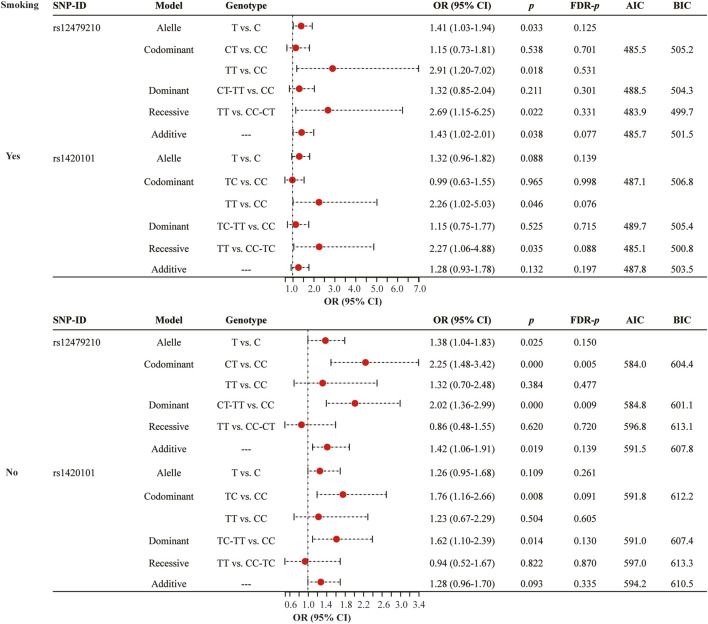
Association between rs12479210 and rs1420101 and lung cancer risk stratified by smoking status. SNP, single nucleotide polymorphism; OR, odds ratio; CI, confidence interval; FDR, false discovery rate; AIC, Akaike Information Criterion; BIC, Bayesian Information Criterion; *p* < 0.05 indicates statistical significance.

Smoking status stratified analysis (Figure 3 and Supplementary Table S2) indicated that rs12479210 (CT vs. CC: OR = 2.25, 95% CI: 1.48-3.42, *p* < 0.0001, FDR-*p* = 0.005; CT-TT vs. CC: OR = 2.02, 95% CI: 1.36-2.99, *p* < 0.0001, FDR-*p* = 0.009) was associated with an increased risk of lung cancer in somking subjects. However, no significant association was found between *IL1RL1* polymorphisms (rs12479210 and rs1420101) and risk of lung cancer in non-smoking. No significant association was found between the four SNPs (rs3771180, rs3771175, rs10208293, and rs10197862) in *IL1RL1* and risk of lung cancer in stratified analysis (Supplementary Table S2).

### MDR analysis

**FIGURE 4:**
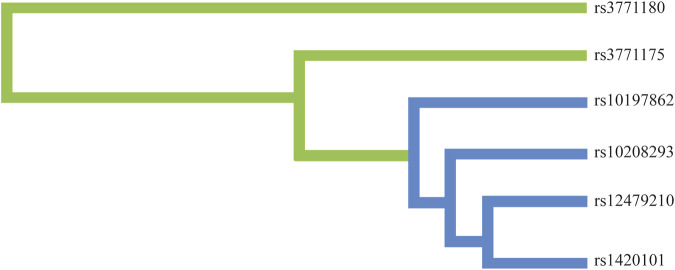
The dendrogram of SNP-SNP interactions.

Moreover, we conducted the MDR analysis to assess the impact of SNP-SNP interactions on risk of lung cancer. The dendrogram of SNP-SNP interactions is displayed in Figure 4, and the results showed a strong negative correlation between rs12479210 and rs1420101 in terms of their impact on lung cancer risk. Moreover, the one loci model (rs12479210) was the best single model with the highest testing accuracy (0.566) and CVC (10/10) to predict lung cancer risk (Table 6).

**TABLE 6:** The impact of SNP-SNP interactions on risk of LC.

Model	Training bal. Acc	Testing bal. Acc	CVC	OR (95% CI)	*p*
rs12479210	0.566	0.566	10/10	1.73 (1.34–2.22)	<0.0001
rs12479210,rs10197862	0.569	0.565	7/10	1.77 (1.38–2.28)	<0.0001
rs12479210,rs10208293,rs10197862	0.573	0.553	5/10	1.85 (1.43–2.40)	<0.0001
rs12479210,rs3771180,rs10208293,rs10197862	0.575	0.550	6/10	1.88 (1.46–2.44)	<0.0001
rs12479210,rs3771180,rs3771175,rs10208293,rs10197862	0.577	0.547	9/10	1.91 (1.48–2.47)	<0.0001
rs12479210,rs3771180,rs1420101,rs3771175,rs10208293,rs10197862	0.577	0.544	10/10	1.91 (1.48–2.47)	<0.0001

LC, lung cancer; Bal. Acc., balanced accuracy; CVC, cross-validation consistency; OR, odds ratio; CI, confidence interval.

*p* < 0.05 indicates statistical significance.

### Bioinformatics analysis

**FIGURE 5:**
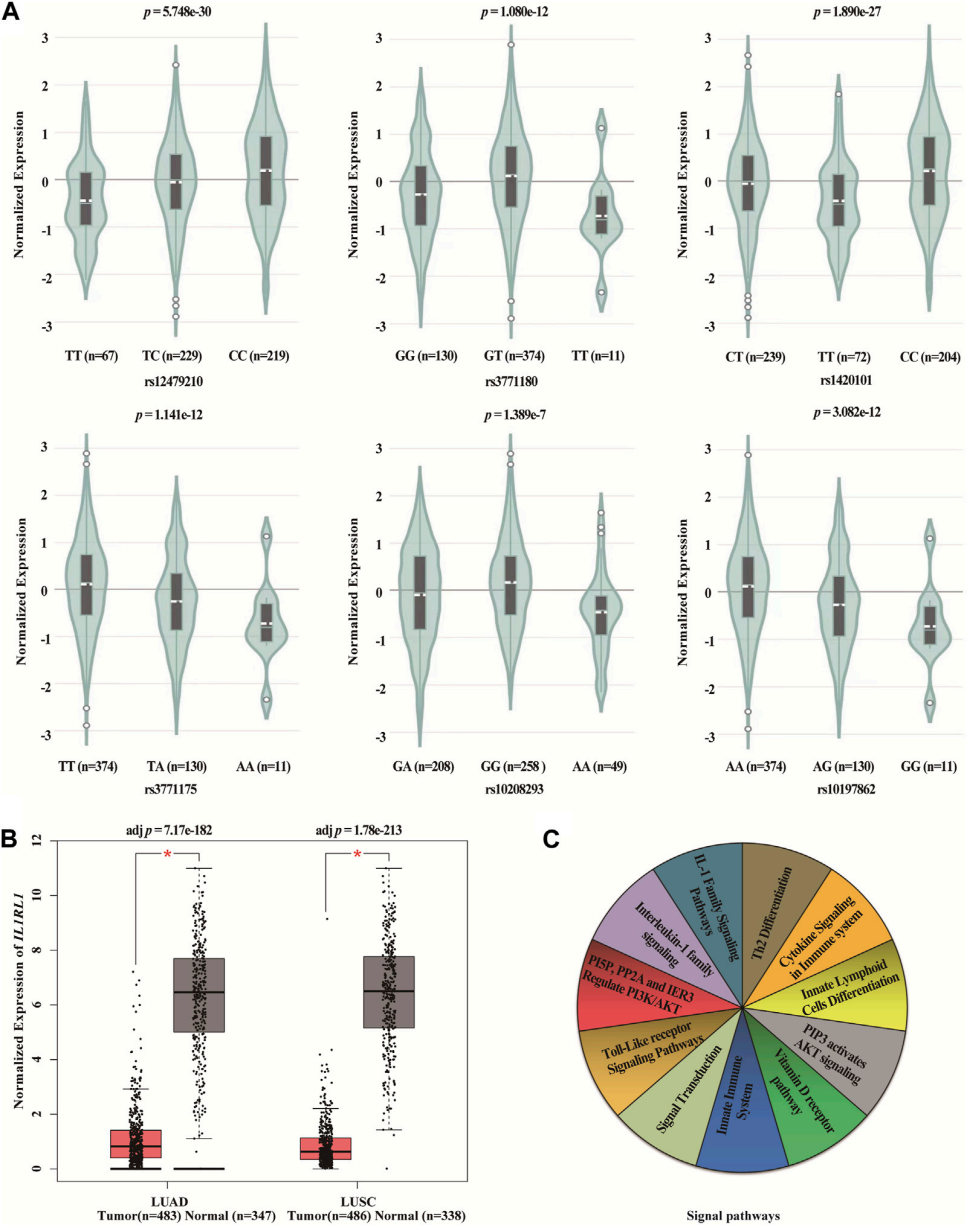
Bioinformatics analysis of *IL1RL1* and polymorphisms **(A)** The differences between six SNPs in *IL1RL1* and the expression level of IL1RL1 in lung cancer tissues. **(B)** The expression level of *IL1RL1* in normal and LUAD and LUSC tissues. **(C)** The signaling pathways involved in the *IL1RL1* gene. LUAD, lung adenocarcinoma; LUSC, lung squamous cell carcinoma; *p < 0.001, *p* < 0.05 indicates statistical significance.

We found statistically significant differences between six SNPs in *IL1RL1* and the expression level of *IL1RL1* in lung cancer tissues using GTEx Portal database (*p* < 0.001, Figure 5A). In addition, we found that the expression level of *IL1RL1* was significantly different between normal and LUAD and LUSC tissues (*p* < 0.001, Figure 5B). We predicted the signaling pathways involved in the *IL1RL1* gene through the PathCards database (Figure 5C), and found that *IL1RL1* was mainly involved in 11 signaling pathways, such as *IL-1* family signaling pathways, Th2 differentiation, cytokine signaling in immune system, and innate lymphoid cells differentiation.

## Discussion

This study is the first to explore *IL1RL1* polymorphisms (rs12479210, rs3771180, rs1420101, rs3771175, rs10208293, and rs10197862) and lung cancer susceptibility in the Chinese Han population. The results showed that rs12479210 and rs1420101 in *IL1RL1* were associated with an increased risk of lung cancer in the Chinese Han population. Moreover, bioinformatics analysis results revealed that the expression level of *IL1RL1* was significantly different between normal and LUAD and LUSC tissues, and there were significant differences between six SNPs in *IL1RL1* and the expression level of *IL1RL1* in lung cancer tissues. These results suggest that *IL1RL1* may be involved in the pathogenesis of lung cancer.

The *IL1RL1* gene is located on chromosome 2q12.1 and contains 13 exons, also known as *IL33R*, *ST2*, and *ST2L*. An animal study has illustrated that *IL1RL1* deficiency could reduce lung myeloid cell infiltration and repeatedly activate macrophage function (Dagher et al., 2020). Liu et al. have revealed that hypoxia significantly increased *IL1RL1* expression by human pulmonary arterial endothelial cells *in vitro* (Liu et al., 2018). The expression levels of *IL-33* and *ST2* were found to be significantly downregulated in both adenocarcinoma and squamous cell carcinoma of the lung compared to adjacent normal lung tissues (Yang et al., 2018). The secretion of *ST2* inhibited tumor growth of lung cancer patients, and low *ST2* was associated with worse overall survival (Tzeng et al., 2018; Gezelius et al., 2022). *sST2*, a soluble form of *ST2*, has been shown to involve in the inflammatory tumor microenvironment and the progression of non-small cell lung cancer (Hong et al., 2019; Chang et al., 2020). A prvious study found that the *IL-33*/*ST2* axis enhances lung-resident CD14^+^ monocyte function in patients with non-small cell lung cancer (Wang et al., 2023). In this study, the expression level of *IL1RL1* was found to be significantly different between normal and LUAD and LUSC tissues based on TCGA database, and *IL1RL1* was mainly involved in IL-1 family signaling pathways, cytokine signaling in immune system, and innate lymphoid cells differentiation. However, this study did not explore the specific mechanism of *IL1RL1* in the occurrence and development of lung cancer.

Furthermore, *IL1RL1* genetic variants have been reported to be associated with susceptibility to respiratorydiseases. A genome-wide association study indicated that rs13431828 and rs1041973 in *IL1RL1* were associated with childhood asthma susceptibility in the Mexican population (Wu et al., 2010). Faber et al. found a genetic association between rs1921622 in *IL1RL1* and disease severity in respiratory syncytial virus bronchiolitis (Faber et al., 2012). *IL1RL1* polymorphisms rs72823628, rs950881 and rs3771175 were found to be associated with a reduced risk of allergic rhinitis risk in the Chinese Han population (Li et al., 2022). However, there are few studies on the correlation between *IL1RL1* polymorphisms and lung cancer susceptibility. In this study, we explore the association betweewn *IL1RL1* polymorphisms and lung cancer susceptibility in the Chinese Han population. This study reports for the first time a significant correlation between *IL1RL1* polymorphisms (rs12479210 and rs1420101) and risk of lung cancer in the Chinese Han population. A previous study indicated that *IL1RL1* rs1420101 was associated with high exhaled nitric oxide and blood eosinophil differentials among Japanese patients with asthma (Inoue et al., 2017). In addition, rs1420101 was strongly associated with *IL1RL1* methylation and serum *IL1RL1-a* levels in asthma (Dijk et al., 2018). Bioinformatics analysis results revealed that there were significant differences between rs12479210 and rs1420101 and the expression level of *IL1RL1* in lung cancer tissue. Therefore, we speculateed that these two SNPs (rs12479210 and rs1420101) may affect the risk of lung cancer by regulating the expression of *IL1RL1*.

In addition, smoking is indisputably linked to lung cancer, yet only a small fraction of smokers develops this disease (Schuller, 2019). A recent report showed that women have a higher risk of developing lung cancer upon smoking than men, and the odds ratio to develop lung cancer was almost three times greater for women than for men (Stapelfeld et al., 2020). The aging of the population is also one of the main reasons for the rise of the incidence rate of lung cancer (Jakobsen et al., 2021). The results of stratification analysis have shown that rs12479210 was related to the susceptibility of lung cancer in different subgroups, while rs1420101 was not significantly associated with lung cancer risk in males, age >60 years old, and smokers. Therefore, we speculated that the impacts of age, gender, and smoking on the susceptibility to lung cancer were stronger than rs1420101. These genetic variants of *IL1RL1* may provide new biomarkers for early diagnosis of lung cancer, and it helps us to have a deeper understanding of the pathogenesis of lung cancer.

Of course, this study has some limitations. First, the significance of *IL1RL1* polymorphisms and lung cancer susceptibility was only performed in the Chinese Han population. There are differences in genetic polymorphisms among different ethnic groups, a larger sample size case-control study can be conducted to further confirm the results of this study in different populations. Second, the occurrence of lung cancer is closely related to environmental factors, but this study lacks some information on BMI and alcohol consumption. Third, lung cancer is a heterogeneous disease, and different subtypes of lung cancer may be associated with different genetic variations. Therefore, in future research, we will collect more comprehensive data, including BMI and alcohol consumption, lung cancer subtypes, etc., to better evaluate the association between *IL1RL1* polymorphisms and lung cancer risk. Finally, the specific molecular mechanism of *IL1RL1* genetic variants in the occurrence and development of lung cancer risk is unclear. Therefore, *in vitro* experiments or animal models can be used to investigate the impact of these genetic variants on *IL1RL1* expression and cell proliferation, apoptosis, and other biological processes. Moreover, the distribution of these genetic variants in lung cancer patients can be analyzed and correlated with clinical data such as tumor type, staging, treatment response, etc., to assess their potential clinical utility.

## Conclusion

In conclusion, this study suggests that *IL1RL1* genetic variants (rs12479210 and rs1420101) contribute to the susceptibility to lung cancer in the Chinese Han population. These risk variants may be used as biomarkers for the prevention and treatment of lung cancer. However, further research is needed to confirm these findings and to better understand the molecular mechanisms underlying the association between *IL1RL1* polymorphisms and lung cancer risk.

## Data Availability

The original contributions presented in the study are included in the article/Supplementary Material, further inquiries can be directed to the corresponding author.

## Abbreviations

AIC, Akaike Information Criterion;; BIC, Bayesian Information Criterion; BMI, body mass index; CHB, Chinese Han Beijing; CI, confidence interval; FDR, false discovery rate; FPRP, false positive report probability; GTEx, Genotype-Tissue Expression; HWE, Hardy-Weinberg equilibrium; IL1RL1, interleukin 1 receptor like 1; LUAD, lung adenocarcinoma; LUSC, lung squamous cell carcinoma; MAF, minor allele frequency; MDR, multifactor dimensionality reduction; OR, odds ratio; SNPs, single nucleotide polymorphisms.

## References

[B1] Bade B. C. Dela Cruz C. S. (2020). Lung cancer 2020: epidemiology, etiology, and prevention. Clin. chest Med. 41 (1), 1–24. 10.1016/j.ccm.2019.10.001 32008623

[B2] Chang C. P. Hu M. H. Hsiao Y. P. Wang Y. C. (2020). ST2 signaling in the tumor microenvironment. Adv. Exp. Med. Biol. 1240, 83–93. 10.1007/978-3-030-38315-2_7 32060890

[B3] Chongtham J. Pandey N. Sharma L. K. Mohan A. Srivastava T. (2022). SNP rs9387478 at ROS1-DCBLD1 locus is significantly associated with lung cancer risk and poor survival in Indian population. Asian Pac. J. cancer Prev. APJCP 23 (10), 3553–3561. 10.31557/APJCP.2022.23.10.3553 36308382 PMC9924343

[B4] Dagher R. Copenhaver A. M. Besnard V. Berlin A. Hamidi F. Maret M. (2020). IL-33-ST2 axis regulates myeloid cell differentiation and activation enabling effective club cell regeneration. Nat. Commun. 11 (1), 4786. 10.1038/s41467-020-18466-w 32963227 PMC7508874

[B5] Dijk F. N. Xu C. Melén E. Carsin A. E. Kumar A. Nolte I. M. (2018). Genetic regulation of IL1RL1 methylation and IL1RL1-a protein levels in asthma. Eur. Respir. J. 51 (3), 1701377. 10.1183/13993003.01377-2017 29519908

[B6] Faber T. E. Schuurhof A. Vonk A. Koppelman G. H. Hennus M. P. Kimpen J. L. (2012). IL1RL1 gene variants and nasopharyngeal IL1RL-a levels are associated with severe RSV bronchiolitis: A multicenter cohort study. PloS one 7 (5), e34364. 10.1371/journal.pone.0034364 22574108 PMC3344820

[B7] Ferreira M. A. McRae A. F. Medland S. E. Nyholt D. R. Gordon S. D. Wright M. J. (2011). Association between ORMDL3, IL1RL1 and a deletion on chromosome 17q21 with asthma risk in Australia. Eur. J. Hum. Genet. EJHG 19 (4), 458–464. 10.1038/ejhg.2010.191 21150878 PMC3060316

[B8] Garrido P. Viñolas N. Isla D. Provencio M. Majem M. Artal A. (2019). Lung cancer in Spanish women: the WORLD07 project. Eur. J. cancer care 28 (1), e12941. 10.1111/ecc.12941 30277293

[B9] Gezelius E. Bendahl P. O. Gallo W. de Oliveira K. G. Ek L. Bergman B. (2022). Circulating levels of the cardiovascular biomarkers ST2 and adrenomedullin predict outcome within a randomized phase III lung cancer trial (RASTEN). Cancers 14 (5), 1307. 10.3390/cancers14051307 35267617 PMC8909619

[B10] Hong J. Kim S. Lin P. C. (2019). Interleukin-33 and ST2 signaling in tumor microenvironment. J. interferon and cytokine Res. 39 (1), 61–71. 10.1089/jir.2018.0044 30256696 PMC6350413

[B11] Huang Y. Zhu M. Ji M. Fan J. Xie J. Wei X. (2021). Air pollution, genetic factors, and the risk of lung cancer: A prospective study in the UK biobank. Am. J. Respir. Crit. care Med. 204 (7), 817–825. 10.1164/rccm.202011-4063OC 34252012

[B12] Inoue H. Ito I. Niimi A. Matsumoto H. Oguma T. Tajiri T. (2017). Association of interleukin 1 receptor-like 1 gene polymorphisms with eosinophilic phenotype in Japanese adults with asthma. Respir. Investig. 55 (6), 338–347. 10.1016/j.resinv.2017.08.006 29153414

[B13] Jakobsen E. Olsen K. E. Bliddal M. Hornbak M. Persson G. F. Green A. (2021). Forecasting lung cancer incidence, mortality, and prevalence to year 2030. BMC cancer 21 (1), 985. 10.1186/s12885-021-08696-6 34479490 PMC8414713

[B14] Khadhraoui C. Kaabachi W. Tritar F. Daghfous H. Hamzaoui K. Hamzaoui A. (2020). Association of BTLA rs1982809 polymorphism with lung cancer risk in Tunisian population. Int. J. immunogenetics 47 (6), 554–562. 10.1111/iji.12491 32757486

[B15] Li Z. Ren J. Zhang J. Wang X. Liu Y. Wang Q. (2022). Association between IL1RL1 gene polymorphisms and allergic rhinitis risk in the Chinese Han population. J. Clin. laboratory analysis 36 (11), e24747. 10.1002/jcla.24747 PMC9701900 36310516

[B16] Liu J. Wang W. Wang L. Chen S. Tian B. Huang K. (2018). IL-33 initiates vascular remodelling in hypoxic pulmonary hypertension by up-regulating HIF-1α and VEGF expression in vascular endothelial cells. EBioMedicine 33, 196–210. 10.1016/j.ebiom.2018.06.003 29921553 PMC6085568

[B17] Malhotra J. Malvezzi M. Negri E. La Vecchia C. Boffetta P. (2016). Risk factors for lung cancer worldwide. Eur. Respir. J. 48 (3), 889–902. 10.1183/13993003.00359-2016 27174888

[B18] Moayedi-Nia S. Pasquet R. Siemiatycki J. Koushik A. Ho V. (2022). Occupational exposures and lung cancer risk-an analysis of the CARTaGENE study. J. Occup. Environ. Med. 64 (4), 295–304. 10.1097/JOM.0000000000002481 35019894

[B19] Rojo-Tolosa S. Sánchez-Martínez J. A. Pineda-Lancheros L. E. Gálvez-Navas J. M. González-Gutiérrez M. V. Jiménez-Gálvez G. (2023). Influence of genetics on the response to omalizumab in patients with severe uncontrolled asthma with an allergic phenotype. Int. J. Mol. Sci. 24 (8), 7029. 10.3390/ijms24087029 37108192 PMC10139019

[B20] Schuller H. M. (2019). The impact of smoking and the influence of other factors on lung cancer. Expert Rev. Respir. Med. 13 (8), 761–769. 10.1080/17476348.2019.1645010 31311354

[B21] Shrine N. Portelli M. A. John C. Soler Artigas M. Bennett N. Hall R. (2019). Moderate-to-severe asthma in individuals of European ancestry: A genome-wide association study. Lancet Respir. Med. 7 (1), 20–34. 10.1016/S2213-2600(18)30389-8 30552067 PMC6314966

[B22] Stapelfeld C. Dammann C. Maser E. (2020). Sex-specificity in lung cancer risk. Int. J. cancer 146 (9), 2376–2382. 10.1002/ijc.32716 31583690

[B23] Sun Y. Wei X. Deng J. Zhang J. He Z. Yang M. (2019). Association of IL1RL1 rs3771180 and TSLP rs1837253 variants with asthma in the Guangxi Zhuang population in China. J. Clin. laboratory analysis 33 (6), e22905. 10.1002/jcla.22905 PMC6642302 31066119

[B24] Sung H. Ferlay J. Siegel R. L. Laversanne M. Soerjomataram I. Jemal A. (2021). Global cancer statistics 2020: GLOBOCAN estimates of incidence and mortality worldwide for 36 cancers in 185 countries. CA a cancer J. Clin. 71 (3), 209–249. 10.3322/caac.21660 33538338

[B25] Taucher E. Mykoliuk I. Lindenmann J. Smolle-Juettner F. M. (2022). Implications of the immune landscape in COPD and lung cancer: smoking versus other causes. Front. Immunol. 13, 846605. 10.3389/fimmu.2022.846605 35386685 PMC8978964

[B26] Tzeng H. T. Su C. C. Chang C. P. Lai W. W. Su W. C. Wang Y. C. (2018). Rab37 in lung cancer mediates exocytosis of soluble ST2 and thus skews macrophages toward tumor-suppressing phenotype. Int. J. Cancer 143 (7), 1753–1763. 10.1002/ijc.31569 29717487

[B27] Wacholder S. Chanock S. Garcia-Closas M. El Ghormli L. Rothman N. (2004). Assessing the probability that a positive report is false: an approach for molecular epidemiology studies. J. Natl. Cancer Inst. 96 (6), 434–442. 10.1093/jnci/djh075 15026468 PMC7713993

[B28] Wang L. Mei X. Liu X. Guo L. Yang B. Chen R. (2023). The interleukin-33/ST2 Axis enhances lung-resident CD14+ monocyte function in patients with non-small cell lung cancer. Immunol. Investig. 52 (1), 67–82. 10.1080/08820139.2022.2130075 36218388

[B29] Wei X. Zhu C. Ji M. Fan J. Xie J. Huang Y. (2021). Diet and risk of incident lung cancer: A large prospective cohort study in UK biobank. Am. J. Clin. Nutr. 114 (6), 2043–2051. 10.1093/ajcn/nqab298 34582556

[B30] Wu H. Romieu I. Shi M. Hancock D. B. Li H. Sienra-Monge J. J. (2010). Evaluation of candidate genes in a genome-wide association study of childhood asthma in Mexicans. J. allergy Clin. Immunol. 125 (2), 321–327. 10.1016/j.jaci.2009.09.007 19910030 PMC2823974

[B31] Wu M. Zheng X. Huang J. Hu X. (2021). Association of IL33, IL1RL1, IL1RAP polymorphisms and asthma in Chinese han children. Front. Cell Dev. Biol. 9, 759542. 10.3389/fcell.2021.759542 34977013 PMC8714920

[B32] Xia C. Dong X. Li H. Cao M. Sun D. He S. (2022). Cancer statistics in China and United States, 2022: profiles, trends, and determinants. Chin. Med. J. 135 (5), 584–590. 10.1097/CM9.0000000000002108 35143424 PMC8920425

[B33] Yang M. Feng Y. Yue C. Xu B. Chen L. Jiang J. (2018). Lower expression level of IL-33 is associated with poor prognosis of pulmonary adenocarcinoma. PloS one 13 (3), e0193428. 10.1371/journal.pone.0193428 29499051 PMC5834175

[B34] Zhang C. Cheng Y. Chen W. Li Q. Dai R. Wang Y. (2022). Association of CYP19A1 rs28757157 polymorphism with lung cancer risk in the Chinese Han population. World J. Surg. Oncol. 20 (1), 400. 10.1186/s12957-022-02868-9 36527059 PMC9756459

