# Supplementary material for: IL1RL1 polymorphisms rs12479210 and rs1420101 are associated with increased lung cancer risk in the Chinese Han population
Source: Front Genet. 2023 Aug 31;14:1183528. doi: 10.3389/fgene.2023.1183528 (PMC10500304; doi:10.3389/fgene.2023.1183528)
Supplement: Supplementary file 2 [file Table1.DOCX]

**Supplemental Table 1 T**he information of six SNPs in *IL1RL1* in the CHB population

| SNP-ID | REF | ALT | MAF | ObsHET | PredHET | HWE-*p* |
| --- | --- | --- | --- | --- | --- | --- |
| rs12479210 | C | T | 0.379 | 0.466 | 0.471 | 1.000 |
| rs3771180 | G | T | 0.097 | 0.155 | 0.175 | 0.464 |
| rs1420101 | C | T | 0.374 | 0.456 | 0.468 | 0.920 |
| rs3771175 | T | A | 0.087 | 0.136 | 0.159 | 0.324 |
| rs10208293 | G | A | 0.146 | 0.194 | 0.249 | 0.073 |
| rs10197862 | A | G | 0.097 | 0.155 | 0.175 | 0.464 |

CHB: Chinese Han Beijing; SNP: single nueleotide polymorphism; REF: Reference allele; ALT: Alternative allele; MAF: minor allele frequency; ObsHET: observed heterozygosity; PredHET:predicted heterozygosity; HWE: Hardy-Weinberg equilibrium

**Supplemental Table 2** Stratified analysis of the association between other four SNPs in *IL1RL1* and LC risk

| SNP-ID | Model | Genotype | OR (95% CI) | *p* | FDR-*p* | AIC | BIC | OR (95% CI) | *p* | FDR-*p* | AIC | BIC |
| --- | --- | --- | --- | --- | --- | --- | --- | --- | --- | --- | --- | --- |
| Gender | | | Male | | | | | Female | | | | |
| rs3771180 | Alelle | T vs. G | 0.92 (0.63-1.35) | 0.661 | 0.991 |  |  | 0.91 (0.54-1.53) | 0.710 | 1.278 |  |  |
|  | Codominant | GT vs. GG | 0.98 (0.65-1.49) | 0.931 | 0.958 | 973.1 | 991.2 | 0.96 (0.54-1.71) | 0.889 | 1.186 | 444.3 | 459.3 |
|  |  | TT vs. GG | 0.32 (0.03-3.11) | 0.326 | 0.839 |  |  | 0.50 (0.04-5.54) | 0.569 | 1.862 |  |  |
|  | Dominant | GT-TT vs. GG | 0.95 (0.63-1.42) | 0.791 | 0.949 | 972.1 | 985.7 | 0.93 (0.53-1.64) | 0.799 | 1.151 | 442.6 | 453.9 |
|  | Recessive | TT vs. GG-GT | 0.32 (0.03-3.12) | 0.328 | 0.737 | 971.1 | 984.7 | 0.50 (0.04-5.57) | 0.572 | 1.585 | 442.3 | 453.6 |
|  | Additive | --- | 0.91 (0.62-1.35) | 0.651 | 1.065 | 972.0 | 985.6 | 0.91 (0.54-1.53) | 0.713 | 1.221 | 442.5 | 453.8 |
| rs3771175 | Alelle | A vs. T | 1.04 (0.69-1.56) | 0.859 | 0.966 |  |  | 0.90 (0.53-1.54) | 0.701 | 1.401 |  |  |
|  | Codominant | AT vs. TT | 1.09 (0.71-1.69) | 0.694 | 0.999 | 975.1 | 993.2 | 0.96 (0.53-1.74) | 0.886 | 1.227 | 444.3 | 459.3 |
|  |  | AA vs. TT | 0.49 (0.04-5.49) | 0.566 | 1.019 |  |  | 0.50 (0.04-5.54) | 0.569 | 1.706 |  |  |
|  | Dominant | AT-AA vs. TT | 1.07 (0.69-1.64) | 0.770 | 0.990 | 973.5 | 987.1 | 0.92 (0.52-1.66) | 0.792 | 1.188 | 442.6 | 453.9 |
|  | Recessive | AA vs. AT-TT | 0.49 (0.04-5.42) | 0.559 | 1.060 | 973.2 | 986.9 | 0.50 (0.04-5.57) | 0.572 | 1.472 | 442.3 | 453.6 |
|  | Additive | --- | 1.04 (0.69-1.56) | 0.864 | 0.943 | 973.5 | 987.2 | 0.90 (0.53-1.54) | 0.705 | 1.336 | 442.5 | 453.8 |
| rs10208293 | Alelle | A vs. G | 0.93 (0.69-1.27) | 0.660 | 1.032 |  |  | 1.01 (0.64-1.59) | 0.975 | 1.063 |  |  |
|  | Codominant | AG vs. GG | 0.97 (0.68-1.37) | 0.852 | 0.989 | 969.4 | 987.6 | 1.15 (0.68-1.93) | 0.613 | 1.471 | 443.7 | 458.7 |
|  |  | AA vs. GG | 0.63 (0.18-2.28) | 0.486 | 1.028 |  |  | 0.51 (0.09-2.85) | 0.443 | 1.995 |  |  |
|  | Dominant | AG-AA vs. GG | 0.95 (0.67-1.33) | 0.747 | 1.034 | 967.8 | 981.4 | 1.08 (0.65-1.79) | 0.769 | 1.203 | 442.6 | 453.8 |
|  | Recessive | AA vs. GG-AG | 0.64 (0.18-2.29) | 0.493 | 0.985 | 967.4 | 981.0 | 0.49 (0.09-2.75) | 0.421 | 2.166 | 442.0 | 453.2 |
|  | Additive | --- | 0.93 (0.68-1.27) | 0.639 | 1.095 | 967.7 | 981.3 | 1.01 (0.64-1.59) | 0.974 | 1.131 | 442.7 | 453.9 |
| rs10197862 | Alelle | G vs. A | 0.94 (0.64-1.39) | 0.770 | 0.956 |  |  | 1.01 (0.60-1.68) | 0.978 | 1.036 |  |  |
|  | Codominant | GA vs. AA | 1.02 (0.67-1.54) | 0.938 | 0.938 | 968.9 | 987.0 | 1.01 (0.57-1.79) | 0.973 | 1.167 | 444.7 | 459.7 |
|  |  | GG vs. AA | 0.32 (0.03-3.11) | 0.327 | 0.784 |  |  | 1.01 (0.14-7.26) | 0.995 | 1.024 |  |  |
|  | Dominant | GA-GG vs. AA | 0.98 (0.65-1.47) | 0.917 | 0.971 | 968.0 | 981.6 | 1.01 (0.58-1.77) | 0.973 | 1.207 | 442.7 | 453.9 |
|  | Recessive | GG vs. AA-GA | 0.32 (0.03-3.10) | 0.325 | 0.901 | 966.9 | 980.5 | 1.01 (0.14-7.23) | 0.996 | 0.996 | 442.7 | 453.9 |
|  | Additive | --- | 0.94 (0.64-1.39) | 0.763 | 1.018 | 967.9 | 981.5 | 1.01 (0.61-1.68) | 0.974 | 1.096 | 442.7 | 453.9 |
| Age | | | > 60 | | | | | ≤ 60 | | | | |
| rs3771180 | Alelle | T vs. G | 1.12 (0.74-1.71) | 0.581 | 0.615 |  |  | 0.70 (0.44-1.12) | 0.135 | 0.304 |  |  |
|  | Codominant | GT vs. GG | 1.36 (0.86-2.15) | 0.194 | 0.583 | 795.4 | 817.1 | 0.68 (0.41-1.12) | 0.125 | 0.347 | 615.4 | 635.8 |
|  |  | TT vs. GG | 0.24 (0.03-2.23) | 0.212 | 0.476 |  |  | 0.92 (0.06-15.10) | 0.956 | 1.110 |  |  |
|  | Dominant | GT-TT vs. GG | 1.25 (0.80-1.96) | 0.333 | 0.544 | 796.2 | 813.6 | 0.68 (0.41-1.12) | 0.129 | 0.332 | 613.4 | 629.7 |
|  | Recessive | TT vs. GG-GT | 0.23 (0.03-2.13) | 0.197 | 0.546 | 795.1 | 812.5 | 1.01 (0.06-16.47) | 0.994 | 1.023 | 615.7 | 632.1 |
|  | Additive | --- | 1.13 (0.75-1.71) | 0.570 | 0.621 | 796.8 | 814.3 | 0.70 (0.43-1.13) | 0.145 | 0.289 | 613.6 | 629.9 |
| rs3771175 | Alelle | A vs. T | 1.29 (0.83-2.01) | 0.258 | 0.464 |  |  | 0.71 (0.44-1.15) | 0.160 | 0.250 |  |  |
|  | Codominant | AT vs. TT | 1.53 (0.94-2.48) | 0.087 | 0.624 | 796.4 | 818.1 | 0.68 (0.41-1.15) | 0.151 | 0.287 | 615.7 | 636.1 |
|  |  | AA vs. TT | 0.34 (0.03-3.37) | 0.358 | 0.496 |  |  | 0.93 (0.06-15.26) | 0.962 | 1.049 |  |  |
|  | Dominant | AT-AA vs. TT | 1.43 (0.89-2.29) | 0.141 | 0.508 | 796.2 | 813.7 | 0.69 (0.41-1.15) | 0.156 | 0.267 | 613.7 | 630.0 |
|  | Recessive | AA vs. AT-TT | 0.32 (0.03-3.18) | 0.334 | 0.522 | 797.3 | 814.8 | 1.01 (0.06-16.47) | 0.994 | 0.994 | 615.7 | 632.1 |
|  | Additive | --- | 1.29 (0.83-2.01) | 0.251 | 0.476 | 797.1 | 814.5 | 0.71 (0.43-1.16) | 0.174 | 0.241 | 613.9 | 630.2 |
| rs10208293 | Alelle | A vs. G | 1.15 (0.83-1.61) | 0.403 | 0.538 |  |  | 0.74 (0.50-1.10) | 0.135 | 0.286 |  |  |
|  | Codominant | AG vs. GG | 1.29 (0.87-1.90) | 0.206 | 0.493 | 792.8 | 814.5 | 0.76 (0.49-1.18) | 0.218 | 0.281 | 615.1 | 635.5 |
|  |  | AA vs. GG | 0.75 (0.23-2.40) | 0.624 | 0.642 |  |  | 0.30 (0.03-2.92) | 0.300 | 0.372 |  |  |
|  | Dominant | AG-AA vs. GG | 1.23 (0.84-1.79) | 0.280 | 0.480 | 791.6 | 809.0 | 0.74 (0.48-1.14) | 0.167 | 0.241 | 613.8 | 630.2 |
|  | Recessive | AA vs. GG-AG | 0.70 (0.22-2.25) | 0.551 | 0.620 | 792.4 | 809.8 | 0.32 (0.03-3.13) | 0.328 | 0.394 | 614.6 | 631.0 |
|  | Additive | --- | 1.14 (0.82-1.60) | 0.432 | 0.537 | 792.1 | 809.5 | 0.73 (0.48-1.10) | 0.130 | 0.311 | 613.4 | 629.8 |
| rs10197862 | Alelle | G vs. A | 1.22 (0.81-1.84) | 0.349 | 0.502 |  |  | 0.72 (0.46-1.14) | 0.163 | 0.244 |  |  |
|  | Codominant | GA vs. AA | 1.43 (0.90-2.27) | 0.130 | 0.585 | 793.9 | 815.6 | 0.70 (0.42-1.15) | 0.154 | 0.277 | 614.2 | 634.6 |
|  |  | GG vs. AA | 0.51 (0.09-2.83) | 0.440 | 0.528 |  |  | 0.93 (0.06-15.22) | 0.960 | 1.080 |  |  |
|  | Dominant | GA-GG vs. AA | 1.34 (0.86-2.10) | 0.203 | 0.521 | 793.3 | 810.7 | 0.70 (0.43-1.15) | 0.158 | 0.259 | 612.2 | 628.5 |
|  | Recessive | GG vs. AA-GA | 0.48 (0.09-2.68) | 0.405 | 0.520 | 794.2 | 811.6 | 1.01 (0.06-16.49) | 0.994 | 1.052 | 614.2 | 630.5 |
|  | Additive | --- | 1.22 (0.81-1.83) | 0.343 | 0.515 | 794.0 | 811.4 | 0.72 (0.45-1.16) | 0.175 | 0.233 | 612.4 | 628.7 |
| Smoking | | | Yes | | | | | No | | | | |
| rs3771180 | Alelle | T vs. G | 2.07 (1.08-3.98) | 0.027 | 0.265 |  |  | 0.67 (0.42-1.06) | 0.084 | 0.334 |  |  |
|  | Codominant | GT vs. GG | 2.09 (1.06-4.13) | 0.034 | 0.094 | 486.4 | 506.1 | 0.73 (0.43-1.22) | 0.225 | 0.312 | 591.6 | 612.0 |
|  |  | TT vs. GG | --- | --- | --- |  |  | 0.16 (0.02-1.43) | 0.100 | 0.277 |  |  |
|  | Dominant | GT-TT vs. GG | 2.13 (1.08-4.21) | 0.029 | 0.174 | 484.8 | 500.6 | 0.66 (0.40-1.10) | 0.110 | 0.233 | 591.8 | 608.1 |
|  | Recessive | TT vs. GG-GT | --- | --- | --- | 489.3 | 505.1 | 0.16 (0.02-1.50) | 0.110 | 0.247 | 591.0 | 607.4 |
|  | Additive | --- | 2.14 (1.09-4.19) | 0.027 | 0.200 | 484.6 | 500.4 | 0.64 (0.40-1.01) | 0.057 | 0.294 | 590.7 | 607.0 |
| rs3771175 | Alelle | A vs. T | 2.09 (1.06-4.12) | 0.031 | 0.154 |  |  | 0.67 (0.42-1.08) | 0.102 | 0.262 |  |  |
|  | Codominant | AT vs. TT | 2.08 (1.02-4.21) | 0.043 | 0.076 | 484.6 | 504.3 | 0.69 (0.40-1.17) | 0.167 | 0.286 | 594.9 | 615.3 |
|  |  | AA vs. TT | --- | --- | --- |  |  | 0.19 (0.02-1.89) | 0.157 | 0.297 |  |  |
|  | Dominant | AT-AA vs. TT | 2.12 (1.05-4.30) | 0.036 | 0.078 | 483.0 | 498.8 | 0.64 (0.38-1.08) | 0.095 | 0.312 | 594.3 | 610.6 |
|  | Recessive | AA vs. AT-TT | --- | --- | --- | 487.1 | 502.8 | 0.20 (0.02-2.01) | 0.173 | 0.283 | 594.8 | 611.2 |
|  | Additive | --- | 2.13 (1.06-4.27) | 0.033 | 0.111 | 482.8 | 498.5 | 0.63 (0.39-1.02) | 0.060 | 0.269 | 593.5 | 609.8 |
| rs10208293 | Alelle | A vs. G | 1.05 (0.67-1.65) | 0.832 | 0.960 |  |  | 0.96 (0.65-1.41) | 0.837 | 0.861 |  |  |
|  | Codominant | AG vs. GG | 1.00 (0.60-1.67) | 0.991 | 0.991 | 486.5 | 506.1 | 1.10 (0.70-1.72) | 0.682 | 0.767 | 597.3 | 617.7 |
|  |  | AA vs. GG | 1.47 (0.15-14.44) | 0.744 | 0.930 |  |  | 0.40 (0.09-1.76) | 0.227 | 0.303 |  |  |
|  | Dominant | AG-AA vs. GG | 1.02 (0.62-1.68) | 0.944 | 1.012 | 484.6 | 500.3 | 1.02 (0.66-1.58) | 0.913 | 0.913 | 597.0 | 613.4 |
|  | Recessive | AA vs. GG-AG | 1.46 (0.15-14.38) | 0.744 | 0.892 | 484.5 | 500.2 | 0.40 (0.09-1.71) | 0.214 | 0.308 | 595.4 | 611.8 |
|  | Additive | --- | 1.03 (0.65-1.65) | 0.890 | 0.989 | 484.6 | 500.3 | 0.95 (0.64-1.41) | 0.802 | 0.875 | 597.0 | 613.3 |
| rs10197862 | Alelle | G vs. A | 1.96 (1.04-3.69) | 0.034 | 0.102 |  |  | 0.72 (0.46-1.14) | 0.158 | 0.284 |  |  |
|  | Codominant | GA vs. AA | 1.99 (1.03-3.85) | 0.042 | 0.078 | 482.3 | 502.0 | 0.74 (0.44-1.25) | 0.255 | 0.328 | 591.9 | 612.3 |
|  |  | GG vs. AA | --- | --- | --- |  |  | 0.30 (0.05-1.71) | 0.176 | 0.275 |  |  |
|  | Dominant | GA-GG vs. AA | 2.03 (1.05-3.93) | 0.035 | 0.082 | 480.8 | 496.5 | 0.69 (0.42-1.14) | 0.149 | 0.297 | 590.9 | 607.3 |
|  | Recessive | GG vs. AA-GA | --- | --- | --- | 484.9 | 500.6 | 0.32 (0.06-1.79) | 0.195 | 0.292 | 591.2 | 607.5 |
|  | Additive | --- | 2.04 (1.06-3.91) | 0.032 | 0.139 | 480.6 | 496.3 | 0.68 (0.43-1.07) | 0.098 | 0.293 | 590.3 | 606.6 |

LC: lung cancer; SNP: single nucleotide polymorphism; OR: odds ratio; CI: confidence interval; FDR: false discovery rate AIC: Akaike Information Criterion; BIC: Bayesian Information Criterion;

*p* < 0.05 indicate statistical significance.
